# Long-term survival in unfavourable-risk mRCC patients after intratumoral administration of a cell-based allogeneic vaccine

**DOI:** 10.1186/2051-1426-3-S2-P438

**Published:** 2015-11-04

**Authors:** Alex Karlsson-Parra, Anna Laurell, Maria Lönnemark, Einar Brekkan, AnnaCarin Wallgren, Anders Magnusson

**Affiliations:** 1 Dept Immunology, Genetics and Pathology, Uppsala University, Uppsala, Sweden; 2 Dept Oncology, Uppsala University, Uppsala, Sweden; 3 Dept Radiology, Uppsala University, Uppsala, Sweden; 4 Dept Surgery, Uppsala University, Uppsala, Sweden

## Background

Initial data from a Phase I/II study in patients with recently diagnosed metastatic RCC (NCT01525017) demonstrated that intratumoral administration of a cell based allogeneic vaccine adjuvant before nephrectomy was associated with a prominent intratumoral infiltration of CD8+ T cells in a majority of patients and a trend towards prolonged survival was noted (Journal of Clinical Oncology, 2014 ASCO Annual Meeting Abstracts. Vol 32, No 15_suppl,, 2014: 3085)

## Methods

Long-term survival as well as data on tumor response after subsequent TKI treatment has been studied.

## Results

Median overall survival (mOS) for MSKCC intermediate risk patients (n = 5) or high risk patients (n = 6) is still not reached but is currently (08JUL2015) 29.0 and 26.0 months, respectively. Three out of 6 patients who to date have received subsequent treatment with TKIs have experienced an objective tumor response. One sunitinib-responding patient (high risk), exhibited an extensive sarcomatoid transformation of the resected primary tumor. Another patient (high risk) who developed 4 brain metastases 4 months after vaccination responded with a complete disappearance of all 4 brain lesions and is still free from CNS-lesions 21 months after initiation of sunitinib. Both patients exhibited a strong/massive intratumoral infiltration of CD8+ T cells in their removed kidney tumor.

## Conclusions

The current mOS (26.0 months) in high-risk patients has by far surpassed reported mOS of 9.0 months in comparable high-risk patients with recently diagnosed mRCC receiving upfront mono-therapy with sunitinib before nephrectomy. The objective tumor responses seen in two high risk patients after addition of sunitinib is surprising since historical data indicate that brain metastases as well as extensive sarcomatoid transformation are higly resistant to sunitinib treatment. Our findings indicate that intratumoral administration of an off-the-shelf vaccine adjuvant consisting of activated allogeneic DCs induces a CTL-mediated anti-tumor response that may prolong survival in mRCC-patients. Data on patients who have received additional treatment with sunitinib (known to deplete myeloid-derived suppressor cells) indicate a synergistic anti-tumoral effect. A multi-centre Phase II study (NCT02432846) has recently been initiated.

